# Chronic stress prior to pregnancy potentiated long-lasting postpartum depressive-like behavior, regulated by Akt-mTOR signaling in the hippocampus

**DOI:** 10.1038/srep35042

**Published:** 2016-10-19

**Authors:** Baomei Xia, Chang Chen, Hailou Zhang, Wenda Xue, Juanjuan Tang, Weiwei Tao, Ruyan Wu, Li Ren, Wei Wang, Gang Chen

**Affiliations:** 1 Center for Translational Systems Biology and Neuroscience, and Key Laboratory of Integrative Biomedicine for Brain Diseases, School of Basic Biomedical Science, Nanjing University of Chinese Medicine, Nanjing, 210023, China; 2 Faculty of Rehabilitation Science, Nanjing Normal University of Special Education, Nanjing, 210023, China; 3 Encephalopathy department, The Third Affiliated Hospital of Nanjing University of Chinese medicine, Nanjing, 210001, China; 4 School of Psychology, Nanjing University of Chinese Medicine, Nanjing, 210023, China

## Abstract

Postpartum depression (PPD) affects over 10% of new mothers and adversely impacts the health of offspring. One of the greatest risk factors for PPD is prepregnancy stress but the underlying biological mechanism is unknown. Here we constructed an animal model which recapitulated prepregnancy stress induced PPD and tested the role of Akt-mTOR signaling in the hippocampus. Female virgin Balb/c mice received chronic restraint stress, followed by co-housing with a normal male mouse. We found that the chronic stress led to a transient depressive-like condition that disappeared within two weeks. However, prepregnantly stressed females developed long-term postpartum depressive-like (PPD-like) symptoms as indicated by deficient performance in tests of sucrose preference, forced swim, and novelty-suppressed feeding. Chronic stress induced transient decrease in Akt-mTOR signaling and altered expressions of glutamate receptor subunits NR1 and GluR1, in contrast to long-term deficits in Akt-mTOR signaling, GluR1/NR1 ratio, and hippocampal neurogenesis in PPD-like mice. Acute ketamine improved the molecular signaling abnormality, and reversed the behavioral deficits in PPD-like mice in a rapid and persistent manner, in contrast to ineffectiveness by chronic fluoxetine treatment. Taken together, we find that chronic prepregnancy stress potentiates a long-term PPD, in which Akt-mTOR signaling may play a crucial role.

Postpartum depression (PPD) is a serious mental disorder commonly identified as a subtype of major depressive disorder (MDD) which occurs within 4 weeks of childbirth^1^, and affects approximately 10% to 20% of mothers^2^. PPD exerts profound adverse effects on mothers and their infants by disturbing maternal behavior and mother-infant interactions. These disturbances can be disabling or life-threatening^3^,^4^. However, the current understanding of the etiology of PPD is incomplete and the mechanisms remain largely unknown.

For the last decades, a number of studies have identified multiple risk factors predicting PPD, including a positive family history of depression, past history of depression and other psychiatric illness, and stressful life events. Currently, the genetic factors influencing PPD remained elusive. Some studies support the hypothesis that experiencing depressed mood or anxiety during pregnancy are significant predictors of PPD^5^,^6^,^7^. A recent study demonstrated that elevated depressed mood during pregnancy is strongly associated with a history of emotional problems (e.g. anxiety and depression) and having experienced stressful events, e.g. the depressed mothers had a history of previous depression more frequently than the non-depressed mothers^8^, indicating that prepregnancy experience of stress or depression can be a substantial factor contributing to PPD. Moreover, it has consistently been demonstrated that depression experienced at any prepregnancy time, not just related to pregnancy or childbirth, significantly increases the risk of PPD, and is one of the largest risk factors for PPD^5^,^6^,^9^. These observations point to the feasible etiological role of prepregnancy experience of stress and depression in PPD. Knowledge of how prepregnancy factors influence PPD is important for improving the strategy toward effective prevention and treatment of the disorder. Until now, however, the role of prepregnancy stress in PPD has not been experimentally assessed. The primary aim of the present study is therefore to test the feasibility using a prepregnancy stress model.

Pharmacotherapy is commonly used for treatment of non-psychotic, mild to severe PPD^10^. Like MDD, about one third of PPD is resistant to chronic treatment of a conventional monoamine-based antidepressant such as fluoxetine, a selective serotonin reuptake inhibitor (SSRI). Recent studies show that a low dose of non-competitive NMDA receptor antagonist, ketamine, is promising for treatment resistant MDD^11^, raising the possibility of using ketamine for treatment resistant PPD. Unlike requirement of chronic administration of conventional SSRIs to elicit an effect, a single dose of ketamine can induce a rapid antidepressant effect, which may last for a week^12^. Additionally, a considerable number of PPD patients display features of bipolar depression^13^, which disqualifies the use of SSRI for the treatment. In light of these concerns, it is necessary to test both monoamine-based and non-monoamine-based antidepressants in a PPD model.

Here, we constructed an animal model of PPD in which the effects of prepregnancy stress on PPD were examined. The effects of chronic treatment using a conventional antidepressant, fluoxetine, and a single dose of ketamine on PPD-like behaviors were also investigated. Emerging evidence has implicated the involvement of Akt signaling in major mental and mood disorders^14^. Several studies demonstrate that the rapid antidepressant effect of ketamine links to the rapid up-regulation of Akt-mTOR signaling^15^,^16^. The Akt-mTOR signaling is compromised in depressed patients and animal models of depression^17^. However, the role of Akt-mTOR pathway in PPD remains unknown. We thus focused on examining the Akt-mTOR signaling pathway, and associated signaling of NMDA receptor subunit NR1 and AMPA receptor subunit GluR1 in the hippocampus. We systematically tested whether alterations of the signaling pathway are associated with short- and long-term depressive-like behavioral phenotypes in females experiencing chronic prepregnancy stress and/or parturition. The molecular and behavioral effects of the acute ketamine or chronic fluoxetine treatment on PPD-like mice were also evaluated. Additionally, the involvement of hippocampal neurogenesis was also assessed. The results showed that chronic prepregnancy stress resulted in a short-term depressive-like condition but potentiated a long-term PPD, which was at least partly attributed to down-regulation of hippocampal Akt/mTOR signaling, neurogenesis and reduced ratio of GluR1/NR1.

## Results

### Chronic stress led to short-term depressive-like conditions and mTOR signaling impairment, but persistent abnormal expression of NR1 and activation of Akt

**Figure 1:**
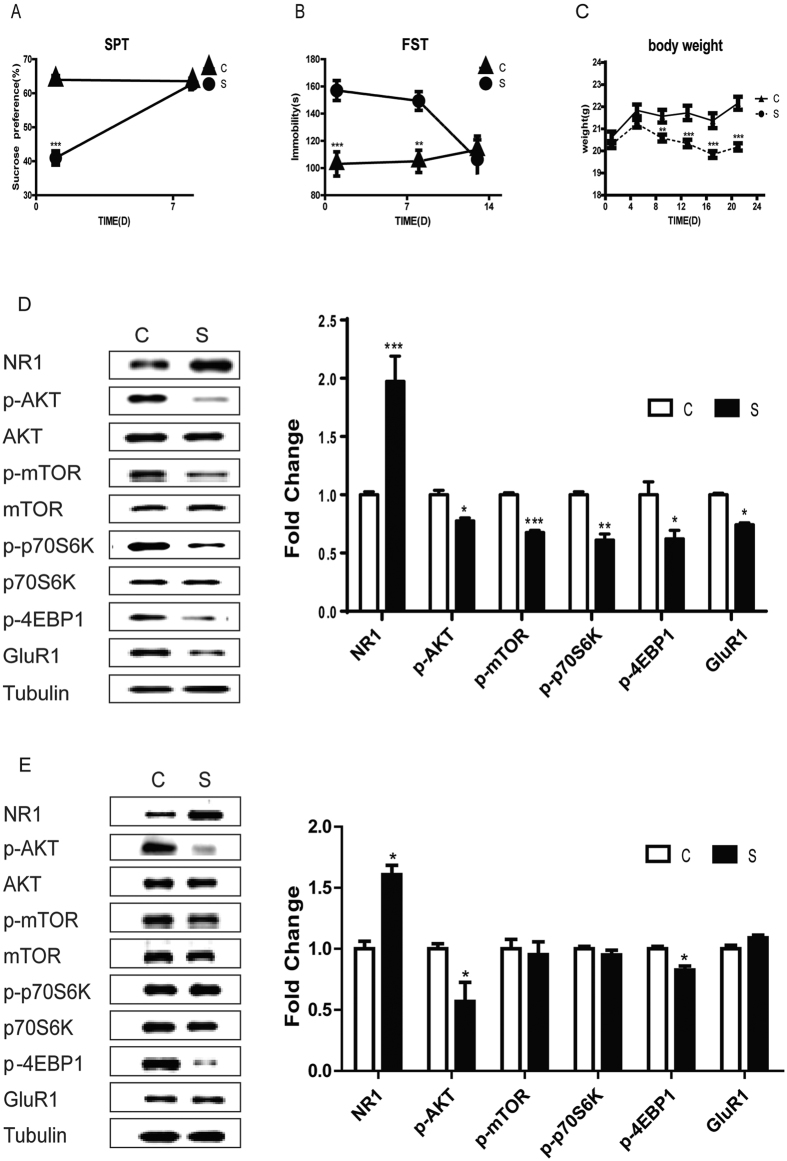
Changes in depressive-like behaviors and Akt/mTOR signaling after chronic stress. (**A**) Sucrose preference test (SPT) and (**B**) Immobility time in the forced swim test (FST) in stressed (S) and control (**C**) groups. (**C**) The effect of stress on the body weight. (**D**,**E**)Hippocampal protein expressions of NR1, Akt, mTOR. p70s6k, 4EBP1, GluR1 1 day (**D**) and 15 days (**E**) after chronic stress. Data represent mean ± SEM. *p < 0.05, **p < 0.01, ***p < 0.001, n = 6–8/group.

Shortly after 3 weeks of chronic stress, virgin female mice exhibited a reduction in sucrose preference (Fig. 1A, t-test, p = 1 × 10^−3^), and increased immobility in the forced swim test (FST) (Fig. 1B, t-test, p = 1 × 10^−3^). Chronic stress significantly decreased weight from day 7 to day 21 (Fig. 1C, t-test, p = 1 × 10^−3^). No significant difference was found in the total distance (OFT; t-test, p = 0.89) and central time in the open field test (OFT; t-test, p = 0.47). However, by 7 days after the last stressor, sucrose preference did not differ between groups (Fig. 1A, t-test, p = 0.56). In contrast, the immobility time in FST was still significantly longer in stressed vs. control mice at 7 days (Fig. 1B, t-test, p = 0.001), but not at 12 days post-stress (Fig. 1B, p = 0.598). Therefore, chronic stress procedure induced a short-term, reversible depressive-like condition.

One day after chronic stress, NR1 protein expression increased significantly in the hippocampus (Fig. 1D, t-test, p = 1 × 10^−3^). Furthermore, chronic stress reduced phosphorylation of Akt (t-test, p = 0.02), mTOR (t-test, p = 1 × 10^−3^), and its effectors, 4EBP1 (t-test, p = 0.012) and p70s6k (t-test, p = 0.01), as well as protein expression of the synaptic protein GluR1 (t-test, p = 0.044). Two weeks after the chronic stress exposure, when the depressive-like phenotype was no longer present (Fig. 1E), NR1 (t-test, p = 0.03) were still up-regulated, and phosphor-Akt and 4EBP1 (t-test, p = 0.043) was still down-regulated. In contrast, mTOR, p70s6k and GluR1 expression in stressed mice did not differ from control mice (t-test, all p > 0.05).

### Chronic prepregnancy stress contributed to long-lasting PPD and dysregulation in Akt-mTOR expression and ratio of GluR1/NR1

**Figure 2:**
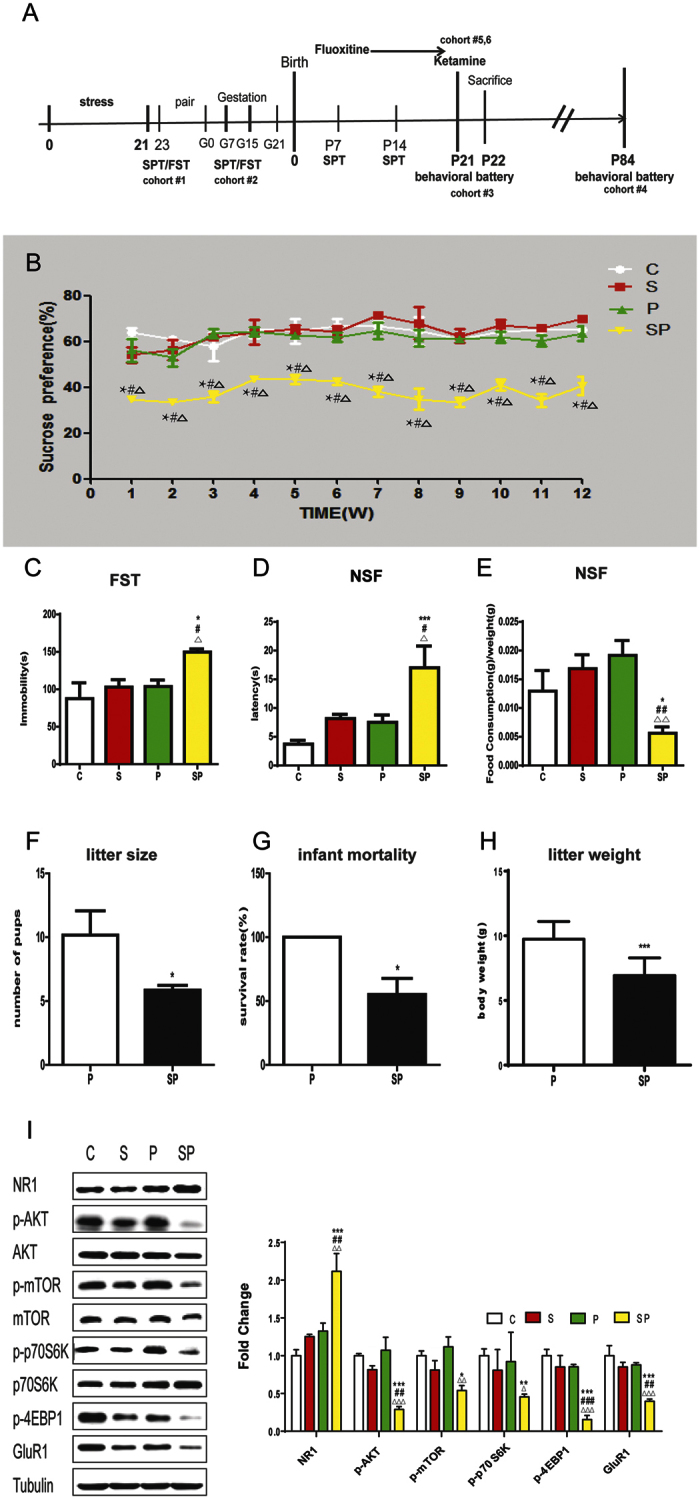
Influences of prepregnancy stress on PPD at 3 weeks postpartum. (**A**) Schematic timeline for experiments. (**B**)SPT from 1 to 12 postpartum weeks in stress-only (S), parturition-only (P), stressed + parturition (SP), and control (C) females. *difference in comparison with C group, ^#^difference in comparison with S group, Δdifference in comparison with P group, *p < 0.01, ^#^p < 0.01, Δp < 0.001,n = 5–7/group. (**C**) Immobility time in FST at 3 weeks postpartum. n = 5–7/group. (**D**,**E**) Latency to take a food pellet (**D**) and food consumption (**E**) in Novelty-suppressed-feeding, NSF test at 3 weeks postpartum. (**F**,**G**,**H**) Effects of prepregnancy stress on the number of pups at birth (**F**) and infant mortality (**G**) and body weights of pups in P and SP groups at 3 weeks postpartum (H). n = 6/group. The results are the mean ± SEM. (**I**) Hippocampal protein expressions of NR1, Akt, mTOR, p70s6k, 4EBP1 and GluR1 at 3 weeks postpartum. n = 5/group. Data represent mean ± SEM. *p < 0.05, **p < 0.01, ***p < 0.001, compared to control group; ^#^p < 0.05, ^##^p < 0.01, ^###^p < 0.001, compared to stress only group; Δp < 0.05, ΔΔp < 0.01, ΔΔΔp < 0.001, compared to parturition group.

To test whether prepregnancy stress influenced PPD, virgin female mice were exposed to 3 weeks chronic stress, followed by co-housing with a male. On average 4 weeks post stress, at a time when behavior in the stressed females did not differ from the control, the females gave birth. A battery of behaviors, including sucrose preference test (SPT), FST, and novelty-suppressed feeding (NSF) test, were assessed at 3 and 12 weeks postpartum, respectively. Three weeks postpartum, only mice experiencing both prepregnancy stress and parturition (giving birth, SP) displayed depressive-like behaviors, compared with stress only (S), parturition only (P) and virgin female control (C) groups. There was a significant reduction in sucrose preference in the SP group [Fig. 2B, *F* (3, 24) = 6.96, p = 0.002]. Immobility time in FST [Fig. 2C, *F* (3, 24) = 3.398, p = 0.039] was significantly increased in the SP group. SP group displayed a significantly increased latency to eat [Fig. 2D, *F* (3, 24) = 8.039, p = 0.001] and decreased food consumption [Fig. 2E, *F* (3, 24) = 4.926, p = 0.010] in the NSF test. In the OFT, there was no effect on the total distance activity [*F* (3, 24) = 1.44, p = 0.259] or time spent in the center field [*F* (3, 24) = 2.148, p = 0.125]. Results from the weekly tests of sucrose preference showed that it was significantly reduced from 1 week postpartum and lasted at least for 12 weeks postpartum [Fig. 2B, repeated measures ANOVA, all p < 0.05]. By 12 weeks postpartum, the deficits were still remarkable in FST [Supplementary Fig1A, *F* (3, 15) = 4.352, p = 0.005] and in NSF [Supplementary Fig1B, for latency to eat, *F* (3, 15) = 16.395, p = 1 × 10^−3^; Supplementary Fig1C, for food consumption, *F* (3, 15) = 22.308, p = 1 × 10^−3^]. There was no significant behavioral difference between C, S and P groups at any surveyed time postpartum. Taken together, these findings indicated a strong contribution of prepregnancy stress to long-lasting PPD. Additionally, the number of offspring born (Fig. 2F, t-test, p = 0.004), survival rate (Fig. 2G, Mann-Whitney test, p = 0.026) and litter weight (Fig. 2H, t-test, p = 1 × 10^−3^) at 3 weeks postpartum was significantly lower in the SP group than P group. There was no significant difference between P and SP group mice in the ratio of female fetuses or ratio of male fetuses (Supplementary 4, t-test, all p > 0.05).

At 3 weeks postpartum, NR1 expression were significantly increased in the SP group in the hippocampus, compared to the S, P and C groups [Fig. 2I, ANOVA, NR1, F(3,19) = 8.994, p = 0.001]. Furthermore, only the SP group showed decreased phosphorylation of Akt, mTOR, 4EBP1, and p70s6k, and decreased expression of GluR1 (AVOVA, all P < 0.05). Both S group and P group showed a trend for increased NR1 expression (AVOVA, p = 0.17).

### A single dose of ketamine ameliorated behavioral deficits and normalized NR1/Akt/mTOR/GluR1 signaling in the PPD-like mice.

**Figure 3:**
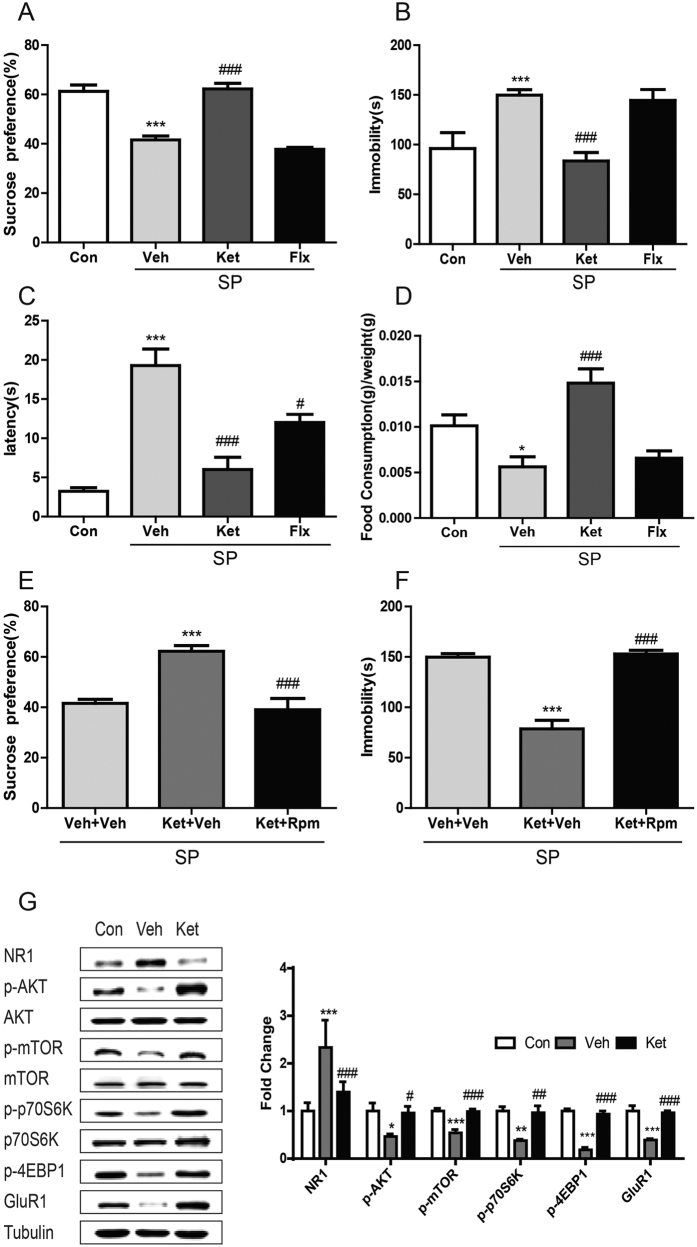
Behavioral effects by acute ketamine or chronic fluoxetine treatment in SP mice and alternations of Akt/mTOR signaling. (**A**,**B**) Effects on the SPT (**A**) or immobility time in FST (**B**) 24 hours after an acute ketamine or 3-week fluoxetine treatment in SP mice. n = 5–7/group.***p < 0.001, compared to control, ^###^p < 0.001, compared to vehicle.(**C**,**D**) Latency to eat (**C**) and food consumption (**D**) in NSF test at 24 hours after an acute administration of ketamine (ket, 30 mg/kg, i.p.) and the last chronic fluoxetine (flx, 18 mg/kg/day x21d, s.c.) treatment in SP mice. n = 5–7/group. *p < 0.05, ***p < 0.001, compared to control, ^#^p < 0.05, ^###^p < 0.001, compared to vehicle. (**E**,**F**) Effects of pharmacological inhibition of mTOR using rapamysin (rapa) on the antidepressant effects of ketamine (ket) on SPT (**E**) and FST (**F**) in SP group mice.n = 8/group.***p < 0.001, compared to non-drug control, ^###^p < 0.001, compared to group of ket + veh. (**G**) Hippocampal protein expressions of NR1, Akt, mTOR, p70s6k, 4EBP1 and GluR1 at 24 hours after an acute administration of ketamine in SP mice, n = 5/group. Data represent mean ± SEM.*p < 0.05, ***p < 0.001, compared to control; ^#^p < 0.05, ^###^p < 0.001, compared to vehicle.

SP group mice received an acute dose of ketamine or chronic fluoxetine treatment. At 3 weeks postpartum, a single dose of ketamine improved the performance of SP group mice in SPT [Fig. 3A, *F* (3, 23) = 36.969, p = 1 × 10^−3^], FST [Fig. 3B, *F* (3, 23) = 12.115, p = 1 × 10^−3^], latency to eat [Fig. 3C, *F* (3,23) = 10.644, p = 1 × 10^−3^], and food consumption [Fig. 3D, *F*(3,23) = 36.995,p = 1 × 10^−3^] in the NSF test compared to vehicle-treated SP group. There was no significant difference in the total distance traveled [*F* (3, 24) = 2.08, p = 0.133] or central time in the OFT [*F* (3, 24) = 0.666, p = 0.582]. The antidepressant effects lasted for at least for 5 days (Supplementary Fig. 3A–D). This was partially mTOR-dependent as pretreatment with mTOR signaling inhibitor rapamycin blunted the antidepressant effects of ketamine on FST [Fig. 3E, *F* (3, 23) = 14.206, p = 1 × 10^−3^] and SPT [Fig. 3F, *F* (3, 23) = 20.109, p = 1 × 10^−3^]. In contrast, chronic fluoxetine treatment for 3 weeks did not improve performance in the SPT, FST, and NSF (Fig. 3A–D) in the SP group, although it was effective on the performance of FST in non-stressed mice [Supplementary Figure 3E, *F*(3,26) = 19.883, p = 1 × 10^−3^]. Acute ketamine reversed the changes in the SP group in hippocampal expression of NR1, Akt-mTOR signaling proteins and GluR1 [Fig. 3G; NR1, *F* (2, 14) = 15.405, p = 1 × 10^−3^; Akt, *F* (2, 14) = 4.205, p = 0.041; mTOR, *F* (2, 14) = 14.634, p = 0.001].

**Figure 4:**
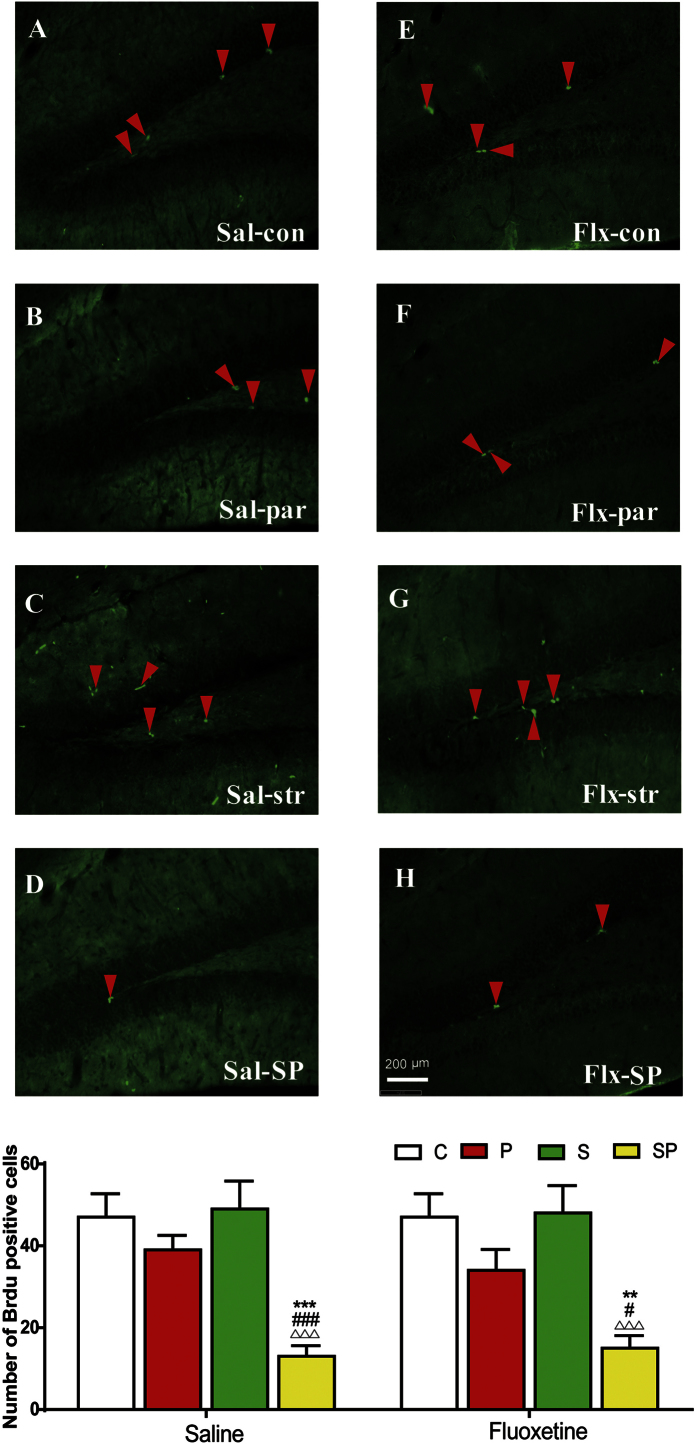
Photomicrographs and quantification of cell proliferation in the hippocampus of PPD-like mice at 3 weeks postpartum and the effects of chronic fluoxetine treatment. BrdU immunoreactivity was detected in the dentate gyrus of hippocampus in control (con, C), stress-only (str, S), parturition-only (par, P) and stress +parturition (SP) groups in the saline (Sal) or fluoxetine (Flx) treatment condition. Left panels (**A**–**D**) represent photomicrographs of saline-treated groups, and right panels (**E**–**H**) represent those of fluoxetine-treated groups. The bottom panel shows the quantification of cell proliferation in each group (I), n = 6/group. Data represent mean ± SEM. **p < 0.01, ***p < 0.001, compared to control group; ^##^p < 0.01, ^###^p < 0.001, compared to stress only group; Δp < 0.05, ΔΔΔp < 0.001, compared to parturition group.

Previous studies show that chronic fluoxetine confers antidepressant effects via promotion of neurogenesis in the hippocampus^18^. Accordingly, we tested whether the hippocampal neurogenesis was influenced by the experimental conditions, using BrdU as the marker for cell proliferation. The number of BrdU-positive cells in C, S and P groups were comparable, but it was dramatically reduced in the SP group, compared to the C, S and P groups [Fig. 4A, ANOVA, *F* (7,47) = 7.136, p = 1 × 10^−3^]. Chronic fluoxetine treatment, however, did not impact the cell proliferation (p > 0.05).

## Discussion

The present study shows that prepregnancy stress and/or short period of depression- like condition contributes to prolonged PPD-like behavior. The debilitating consequence of PPD on offspring was also exemplified^19^. Chronic prepregnancy stress resulted in sustained up-regulation of NR1 expression but a short-term down-regulation of Akt/mTOR signaling in the hippocampus. Mice that experienced chronic prepregnancy stress became vulnerable to postpartum depression, and also exhibited not only an abnormal GluR1/NR1 expression ratio but also a deficit in Akt/mTOR signaling. Additionally, SP mice showed a dramatic reduction in the cell proliferation in the hippocampus. The deficit in NR1/Akt/mTOR signaling and behavioral symptoms in SP mice could be reversed by a single administration of ketamine, but not by chronic fluoxetine administration. These observations suggest that prepregnancy stress induced changes in NR1/Akt/ mTOR/GluR1 signaling may play an important role in regulating PPD-like symptoms.

Several animal models have been developed to study the development of and treatment for PPD. The most widely used PPD model is based on ovarian- steroid- withdrawal^20^, with hypothesis that ovarian hormones, including estrogen and progesterone, are critical in mediating depressive-like symptoms after withdrawal from pregnancy-associated hormones. Disadvantages of the model include its inability to assess the effects of parturition directly and the subsequent effects on the offspring as females were ovariectomized. Emerging models highlight the importance of stress in PPD. For example, gestational stress, pregestational stress or high postpartum corticosterone induced depressive-like behaviors or changes in maternal care postpartum^21^,^22^,^23^. Here, we showed a chronic prepregnancy stress paradigm which led to a short-term depressive-like condition which then potentiated the development of PPD. Three characteristics make this model invaluable for studying PPD. First, long-lasting PPD-like behaviors which persist for at least 3 months postpartum in females chronically-stressed prior to pregnancy; Second, prepregnancy stress had a remarkable impact on the number of offspring, and their survival rate. In fact, the surviving offspring of SP mice showed depressive-like behavioral deficits observable both at juvenile and adulthood^24^. Third, PPD-like mice selectively responded to some but not all antidepressants. The validity of this animal model of PPD can be exploited to test the efficacy of post-intervention with pharmaceutical or behavioral approaches that can eventually lead to remission from PPD symptoms. Further study should evaluate whether the variations in intensity, duration, and severity of the prepregnancy stress used in the present study affects the sexual/gonadal axis, and its link with long-term PPD-like symptoms and/or treatment.

**Figure 5:**
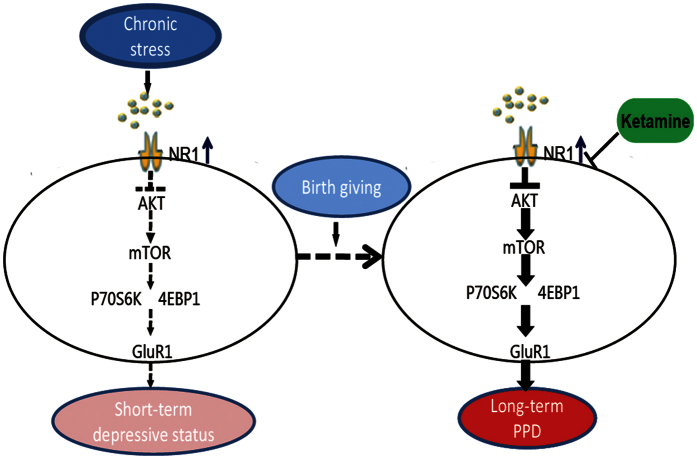
A model of Akt-mTOR signaling pathways in developing PPD potentiated by prepregnancy stress. Chronic mild stress results in up-regulation of NMDA receptor subunit NR1, which suppresses the activation of Akt, in turn down-regulates mTOR signaling in the hippocampus. This abnormal signaling underscores the depressive-like behavior, although the deficit can be gradually restored, responsible for behavioral recovery. With the occurrence of parturition, the chronic stress, however, facilitates a prolonged NR1/Akt/mTOR signaling deficiency and neurogenesis postpartum, subsequently induces a long-term postpartum depression. The defect Akt-mTOR signaling can be corrected by a single dose of ketamine and thus a quick and enduring antidepressant effect is achieved.

Our findings demonstrated that stress induced a dynamic change in expression of the membrane synaptic proteins NR1 and GluR1 as well as intracellular Akt/mTOR signaling. Akt/mTOR signaling can be temporally activated, which in turn leads to long-time up-regulation of synaptic proteins including GluR1 responsible for a rapid antidepressant effects^15^. The present study showed NR1 expression was up-regulated and Akt/mTOR/GluR1 signaling was down-regulated, respectively, shortly after chronic restraint stress, with signs of depressive-like symptoms. Signaling of mTOR/GluR1 was able to be restored at the later time, in correspondence to the normalized behavioral responses. In contrast, there was long-lasting dysregulation in NR1/Akt/mTOR/GluR1 signaling in mice that experienced both prepregnancy stress and parturition, along with long-lasting depressive-like behaviors. Ketamine attenuating NR1 expression and up-regulating Akt/mTOR/GluR1 signaling conferred rapid antidepressant effects, which could be blunted by blockade of mTOR signaling. These findings corroborate results from a recent study demonstrating that a persistent NR1 up-regulation and Akt down-regulation was associated with maintenance of depressive-like responses after chronic mild stress, whereas the reversal of NR1 expression and Akt signaling abnormality by the rapid antidepressant herb medicine Yueju or ketamine linked to antidepressant effects^16^. Therefore, stress induced NR1 and Akt signaling defect may act as the upstream activator to influence mTOR signaling that regulates depressive-like responses. Although other unknown factors may counteract altered NR1 and Akt signaling to normalize mTOR signaling and behavior over time after chronic stress, a subsequent event of parturition potentiates a prolonged deficit in mTOR signaling that is likely responsible for persistent depressive-like behavior, as shown in the present model. This scenario is schematically visualized in Fig. 5. Findings from our group and others warrant further studies on the mechanism by which parturition and prepregnancy stress alter mTOR and other signaling governing depression.

At the present time, there is very few therapeutic regimen that is specific for PPD and it can take a long time for an SSRI to exert antidepressant effects^25^. This can have a remarked developmental impact on infants of PPD mothers. Some SSRIs have side effects, via breastfeeding, on infant’s developing neural systems, and may increase the risk of women’s suicide and/or infanticide^26^. Additionally, meta-analysis shows that about 30% of PPD patients fail to respond satisfactorily to conventional antidepressants, and are classified as treatment-resistant PPD^19^. Our model displayed treatment-resistant-like PPD features based on the observations that chronic fluoxetine treatment was ineffective to some measurements of PPD. Some SSRIs were effective in reversing the depressive-like behavior in certain animal models of PPD and modifying brain areas other than hippocampus. For example, in a gestational stress based PPD model, depressive-like behavior in the forced swim test was effectively treated by citalopram administration, which may link to normalization of structural alterations in the postpartum NAc shell and mPFC^27^. Therefore further study should investigate other antidepressants and brain areas that play a role in PPD. Ketamine has been found effective on treatment-resistant depression patients^28^, and here we showed it rapidly and lastingly alleviated the PPD-like symptoms, raising the possibility of the use of ketamine for patients with treatment-resistant PPD. However, the safety of ketamine on the infant development remains to be established. The safe, fast-onset and effective antidepressants are still urgently needed for the benefits of the PPD mothers and their children. For example, Yueju showed the rapid antidepressant potential^23^,^29^, which also elicited effects similar to ketamine on the current PPD model (Supplementary Fig. 5), and may become an option to fight treatment-resistant PPD clinically.

Accumulating evidence supports the link of hippocampal neurogenesis to depression and antidepressant activity. Here, consistent with the ineffectiveness on behavior by chronic fluoxetine treatment, we found it did not promote the cell proliferation in the hippocampus in virgin females or prepregnancy-stressed parturient females, in agreement with some of the previous reports^30^,^31^. We detected prepregnancy stress in combination of parturition decreased the hippocampal cell proliferation 3 weeks postpartum, which may play a part in PPD behavior. Stress, pregnancy and antidepressants have been shown to differently influence different stages of hippocampal neurogenesis including cell proliferation, immature cells and new surviving cells in females previously. Stress early in gestation increases neurogenesis, compared to non-stressed virgin females^32^. Stress during the late pregnancy reversed the decreased cell proliferation in late pregnant females^33^, but it is not evident in postpartum females one week after weaning^34^. However, we found that prepregnancy stress plus parturition reduced cell proliferation measured at three weeks postpartum, whereas there was not significant effect in stress only virgin females or parturition only females. The differences may be accounted for by various stress models, time courses, genetic backgrounds, *et al.* Interestingly, ketamine was also found to have a long-term impact on hippocampal neurogenesis^35^. Although ketamine-induced neurogenesis is unlikely responsible for the rapid antidepressant response, it may be involved in the persistent antidepressant effects on PPD-like mice.

This study developed a novel animal model to assess PPD-like behavior and associated molecular substrates. However, there are several limitations. First, we focused on assessing PPD using the conventional depressive-like behavioral paradigms. Further studies should test maternal pup-directed behaviors and maternal behavior that represents a maternal specific measure of anhedonia^36^. Second, it is unknown whether this PPD model is strain-dependent or species dependent. Third, the finding of reduced hippocampal neurogenesis in PPD-like mice can be further verified using the endogenous markers of cell proliferation such as ki67^37^. Despite of these limitations, the present study demonstrates for the first time the remarkable contribution of prepregnancy stress to PPD development. The results suggest that a deficit in hippocampal Akt-mTOR signaling as a key pathogenic mechanism. We also demonstrated ketamine can be used to treat treatment-resistant-like PPD symptoms by rapidly normalizing Akt-mTOR signaling. This study sheds a new light on the pathophysiology of PPD and offers new insight for better treatment of the devastating disorder.

## Methods

### Animals

6–8 week old Balb/cJ female and male mice, weighing 18–24 g, were housed in the animal facilities for 1 week before experiments. Mice were kept on a 12 h/12 hr light/dark cycle and were given free access to food and water. All animal procedures conformed to the Guide for the Care and Use of Laboratory Animals and were approved by the Institutional Animal Care and Use Committee at Nanjing University of Chinese medicine. The experimenters were blind to the assignments of the mice.

### Experimental design

A total of 156 naive females were age-matched prior to random assignment to experimental groups. Six cohorts of mice received a three week chronic mild stress procedure (Fig. 1A). In cohorts #1 (n = 16) and #2 (n = 16), female mice were randomly divided into stressed and non-stressed groups. The stressed mice were subjected to daily 6 hr restraint stress in a 50 ml centrifuge tube, combined with overnight illumination twice a week for 3 weeks. All stressed and non-stressed mice were group housed. In cohort #1, animals were tested with a behavioral battery two days after the last stressor. In cohort #2, animals were behaviorally tested 7 and 12 days after the last stressor. The same stress procedure was used in cohort #3–#6. In cohort #3 (n = 48), two days after the last stressor, half of the stressed or non-stressed females were introduced to a normal male, and the other half of the groups were kept in group housed with other female mice. For breeding, two female and one male were housed together in a cage, and the male removed until the vaginal plug was released. The average time to impregnation was 1 week, comparable for stressed and non-stressed female mice. About 3 weeks later, the mated females gave birth.

There were four randomly assigned experimental groups: (1) control group, which did not experience chronic stress and pregnancy(C); (2) stressed only group that did not experience pregnancy (S); (3) parturition only group which did not experience prepregnancy stress (P); and (4) stressed plus parturition group (SP). Female mice in these 4 groups were tested with a battery of depressive-like behaviors at postpartum week 3. In cohort #4 (n = 24), the mice were tested for the behaviors at postpartum week 12. Additionally, the sucrose preference test was carried out weekly from postpartum week 1 to 12. In cohort #5 (n = 24), antidepressants were administered and behaviors were tested at 3 weeks postpartum. The procedure for cohort #6 (n = 24) was similar to cohort #5, except the administration of rapamycin or vehicle at 30 min before ketamine administration.

### Calculation of offspring litter size and survival rate

The number of pups at birth was recorded, starting from postnatal day 0 and continuing until day 21. All litters were weaned at postnatal day 21. The survival rate of the offspring was calculated with the number of pups weaned divided by the number of pups born. The body weight of litters and sex was also recorded on postnatal day 21.

### Behavior Testing

#### Open field test (OFT)

The OFT assesses locomotor activity and anxiety-like behavior in a bright-lit open area. Testing was performed in a well-illuminated (~300 lux) transparent acrylic cage (40 × 40 × 15 cm). The mice were gently placed in the center and left to explore the area for 5 minutes. The digitized image of the path taken by each mouse was tracked by camera, and the total running distance (locomotor activity) and time spent in the center were analyzed using ANY-maze software. Each mouse was placed in the middle of a peripheral zone of the arena facing the wall and allowed to freely explore the apparatus, with the experimenter out of the animal’s sight.

#### Forced swim test (FST)

FST was carried out as reported by Porsolt *et al.*^38^. Each mice was placed into a transparent Plexiglass cylinders (25 cm high, 10 cm in diameter) containing 10 cm of water maintained at 23–25 °C. Animals were left in the cylinder for 6 min. The total duration of immobility was measured during the last 4 min. The animals were considered to be immobile when they floated passively in the water.

#### Sucrose preference test (SPT)

SPT was performed following the procedure of Opal^39^, all mice were trained to consume two bottles of sucrose solution (2%) for 3 days to establish baseline preference levels. After 18 hours of food and water-deprivation, mice were singly housed and presented with two pipettes containing 2% sucrose solution or tap water for 1 h. Sucrose preference was calculated by the formula: (sucrose preference) = ((sucrose intake)/(sucrose intake + water intake)) × 100, as previously described.

#### Novelty suppressed feeding (NSF) test

Twenty-four hours before the NSF test, the mice were deprived of food but not water. On the day of the test, mice were placed at the edge of the plexiglass test chamber (30 cm × 60 cm) with a single food pellet in the center in a quiet room with dim lighting. Latency to feeding was measured for 5 minutes; non-feeding behaviors (e.g, touching, smelling) were ignored. If food was not taken within 5 minutes, feeding latency was regarded as 5 minutes. Food pallets were weighed before and after test.

### Drug administration

Fluoxetine (Sigma-Aldrich, St. Louis, MO, USA) was dissolved in 0.9% saline following the procedure of Dulawa *et al.*^40^. Fluoxetine or saline was administered once daily intraperitoneally (i.p.) for 21 days, starting on postpartum day 1. On postpartum day 21, after the final saline/fluoxetine administration, mice were administered i.p. bromodeoxyuridine (BrdU) (4 × 75 mg/kg, every 2 hours) (Sigma, St. Louis, MO, USA), and then the next morning mice were tested for depressive-like behaviorsand sacrificed at 24 hours after the last BrdU injection. Ketamine HCl (Gutian Pharmaceuticals, China) was dissolved in saline. Ketamine or saline control was administered i.p. at 24 hours before the behavior tests. The drug doses, with the final dose in parentheses, were as follows: fluoxetine (18 mg/kg/day), and ketamine (30 mg/kg). Rapamycin (LC Laboratories) was first dissolved in ethanol at a stock concentration of 25 mg/ml and was diluted for a final concentration of 1 mg/ml in 4% ethanol, 5% Tween 80, and 5% PEG 400 as previously described. Rapamycin or corresponding vehicle was i.p delivered to mice (20 mg/kg) 30 min before ketamine administration.

### Western blot

The whole hippocampus (ventral and dorsal) was lysed in RIPA buffer containing protease inhibitors and phosphatase inhibitors. Protein concentration was determined colorimetrically by NANODROP 2000 (Thermo Scientific). Protein lysates were separated by 10% SDS-PAGE electrophoresis and were transferred onto polyvinylidenedifluoride (PVDF) membranes. After blocking with 5% BSA for 1 hr, and the following antibodies were used: NR1(Santa Cruz Biotechnology, 5704S, 1 : 500), AKT/p-AKT (Cell Signaling Technology, 9272S, 4058s, 1:500), mTOR/p-mTOR (Cell Signaling Technology, 2972S, 2971S,1:500), p70S6 kinase p70s6k) / p--p70S6K (Cell Signaling Technology, 2708S, 9234S, 1:500), p-4EBP1 (Cell Signaling Technology, 9644S.1:1000), GluR1 (Cell Signaling Technology, 12551S, 1:1000) and β-tubulin (Epitomics, 1879-1, 1:2000). After the blots were incubated with antibodies overnight at 4 °C, they were incubated with horseradish peroxidase- conjugated secondary antibodies for 1 hr. The blots were visualized using the SuperSignal West Pico Chemiluminescent Substrate (Thermo Fisher Scientific Inc.). The resulting data was analyzed statistically using SPSS Statistics 20. All experiments were performed in troplicate. The final data are expressed as a ratio of the relative optical density (ROD) of the protein of interest to the ROD of β-tubulin. A p-value < 0.05 was considered significant.

### Immunohistochemistry

The procedure for BrdU immunostaining followed Chen *et al.*^41^ with minor modification. At 24 h after the last BrdU injection, the mice were killed and their hearts perfused with 0.01 M cold phosphate-buffered saline (PBS) for 5 min, followed by 4% cold paraformaldehyde for 20 min. Brains were removed and post-fixed in paraformaldehyde at 4 °C in 30% sucrose. Serial sections were prepared (30 μm sections) through the entire hippocampus on a freezing microtome. One of six parallel brain sections was used for immunostaining. The number of proliferating cells was assessed in the dentate gyrus of the hippocampus using exogenous marker, BrdU. Mounted sections were digested in trypsin (0.001%) in Tris buffer containing 0.1% CaCl_2_ for 10 min, denaturated in 2N HCl for 30 min for BrdU staining pretreatment. After thorough rinses and incubation with blocking solution, sections were then incubated with a primary antibody (anti-mouse BrdU, Millipore, 1:4000 in the blocking solution overnight. On the following day, sections were rinsed, and incubated for one hour with goat anti-mouse IgG Alexa Fluor-488 secondary antibody (Invitrogen, 1:1000), in the bilateral hippocampal region was counted for each mouse. All BrdU-positive cells in the dentate gyrus were counted using microbrightfield stereo investigator software (microbrightfield, Williston,VT).

### Statistical Analyses

Two-sample comparisons were carried out using the two-tailed Student’s t-test, unless otherwise stated; Mann-Whitney test were employed when the data was not normally distributed. Multiple comparisons were made using ANOVA, followed by the Newman-Keuls multiple range test. Univariate and analysis of variance with repeated measures (repeated-measures ANOVA, ANOVA-rm) were also performed for the time-course study of postpartum sucrose preference test, with treatment as the between subject factor and time as the within subject factor. The threshold for statistical significance was set at p < 0.05. All results are indicated as the mean ± SEM.

## Additional Information

**How to cite this article**: Xia, B. *et al.* Chronic stress prior to pregnancy potentiated long-lasting postpartum depressive-like behavior, regulated by Akt-mTOR signaling in the hippocampus. *Sci. Rep.* **6**, 35042; doi: 10.1038/srep35042 (2016).

## Supplementary Material

Supplementary Information
