# Supplementary material for: Chronic stress prior to pregnancy potentiated long-lasting postpartum depressive-like behavior, regulated by Akt-mTOR signaling in the hippocampus
Source: Sci Rep. 2016 Oct 19;6:35042. doi: 10.1038/srep35042 (PMC5069466; doi:10.1038/srep35042)

Chronic stress prior to pregnancy potentiated long-lasting postpartum depressive-like behavior, regulated by Akt-mTOR signaling in the hippocampus

Baomei Xia [1, 2,*], Chang Chen [3,*], Hailou Zhang[1], Wenda Xue[1], Juanjuan Tang[1], Weiwei Tao[1], Ruyan Wu[1], Li Ren[1], Wei Wang [4] and Gang Chen [1]

[1] Center for Translational Systems Biology and Neuroscience, and Key Laboratory of Integrative Biomedicine for Brain Diseases, School of Basic Biomedical Science, Nanjing University of Chinese Medicine, Nanjing 210023, China

[2] Faculty of Rehabilitation Science, Nanjing Normal University of Special Education, Nanjing 210023, China

[3] Encephalopathy department, The Third Affiliated Hospital of Nanjing University of Chinese medicine, Nanjing 210001, China

[4] School of Psychology, Nanjing University of Chinese Medicine, Nanjing 210023, China

*These authors contributed equally to this work. *** Correspondence:** Dr. G. Chen, Professor, Center for Translational Systems Biology and Neuroscience, School of Basic Biomedical Science, Nanjing University of Chinese Medicine, Nanjing 210023, China.

Tel: 86 25 85811160

Fax: 86 25 85811160

E-mail: chengang@njucm.edu.cn

Supplementary

Supplementary 1. The behavioral tests at 12 weeks postpartum.

By 12 weeks postpartum, The SP group mice still demonstrated deficits in FST **(A)**[F (3, 15) = 4.352, p =0.005] and in NSF test, latency to eat **(B)**, [AVONA, F (3, 15) =16.395, p =1x10^{-3}], food consumption **(C)** [AVONA, F (3,15) =22.308 ,p =1x10^{-3}]. The results are the mean ± SEM, ANOVA, n=3-6/group. *p<0.05, ** p<0.01, *** p<0.001. #p<0.05, ## p<0.01, ### p<0.001.Δp<0.05, ΔΔ p<0.01, ΔΔΔ p<0.001.

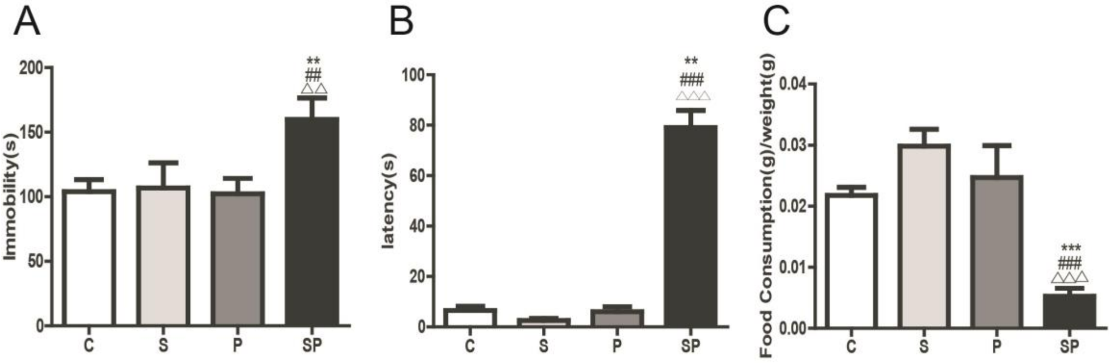

Supplementary 2. Hippocampal protein expressions of NR1, Akt, mTOR, p70s6k, 4EBP1, GluR1 24hr postpartum in control (C) and parturition-only (P) females. The results are the mean ± SEM, ANOVA, n=6/group.

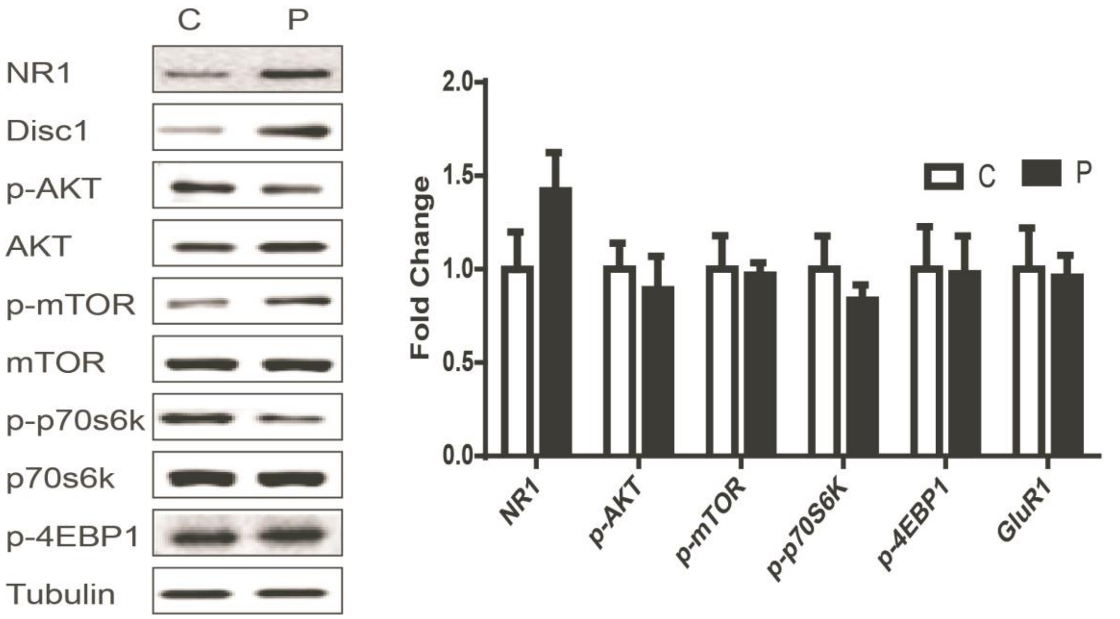

Supplementary 3. The lasting antidepressant-like effects, assessed with SPT, FST and NSF, on the stress + parturition (SP) mice at 5 days after acute ketamine (Ket) and vehicle (Veh). For SPT **(A)**, AVONA, F (2, 11) = 26.421, p =1x10^{-3}, FST **(B)**, AVONA, F (2, 11) = 21.242, p =1x10^{-3}, NSF, latency to eat **(C)**, AVONA,F (2, 11) =53.405, p =1x10^{-3}, food consumption **(D)**, AVONA,F (2, 11) =7.814, p =0.011, n=3-5/group. **(E)**Immobility time in FST in PPD mice and control (Con) mice after chronic fluoxetine. Chronic fluoxetine treatment was effective on the FST in

non-stressed control mice, AVONA, $F(3,26) = 19.883$, $p = 1\times10^{-3}$. The results are the mean ± SEM, ANOVA, n=6-7/group.

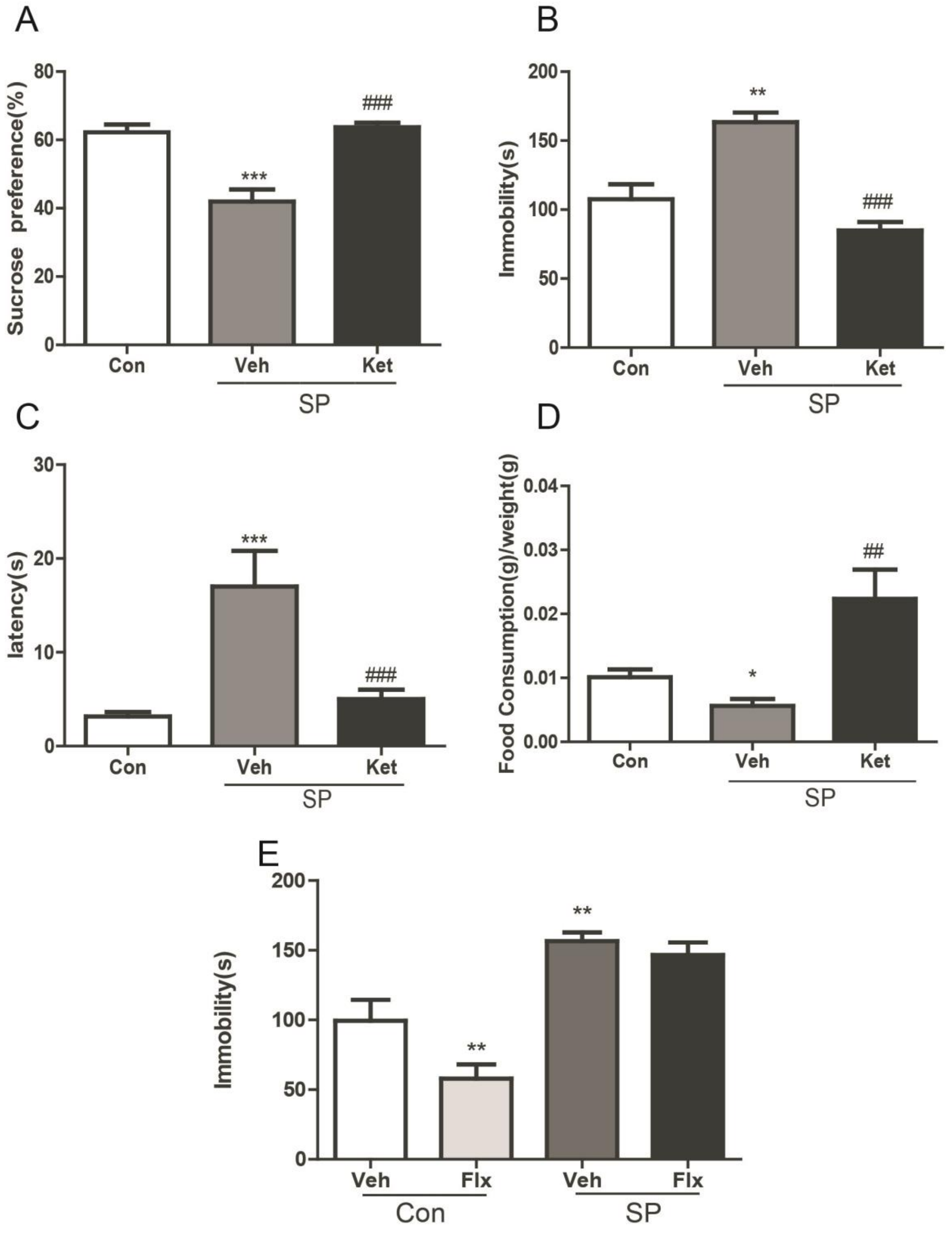

Supplementary 4. The number of female and male fetuses in the P and SP group. n=6, data represent sex ratio. T-test.

	P	SP
Female/total pups(%)	29/52（55.77%）	45/82（54.87%）
Male/total pups(%)	23/52（44.23%）	37/82（45.13%）

Supplementary 5. Antidepressant effects 24 hours after a single Yueju treatment in SP mice. The procedure and dose of Yueju followed Tang et al.[23.] Control animals (Con) received vehicle treatment, and animals exposed to CMS received a single administration of vehicle (Veh) or Yueju (YJ). (A) Sucrose preference test after drug administration. (B) The immobility time in the FST 48 hours post drug administration. (C) Latency to feed and (D) Total amount of food consumed during the NSF test post drug administration. The results are the mean ± SEM, ANOVA, n=6/group. *** p<0.001, compared to control, ### p<0.001, compared to vehicle.

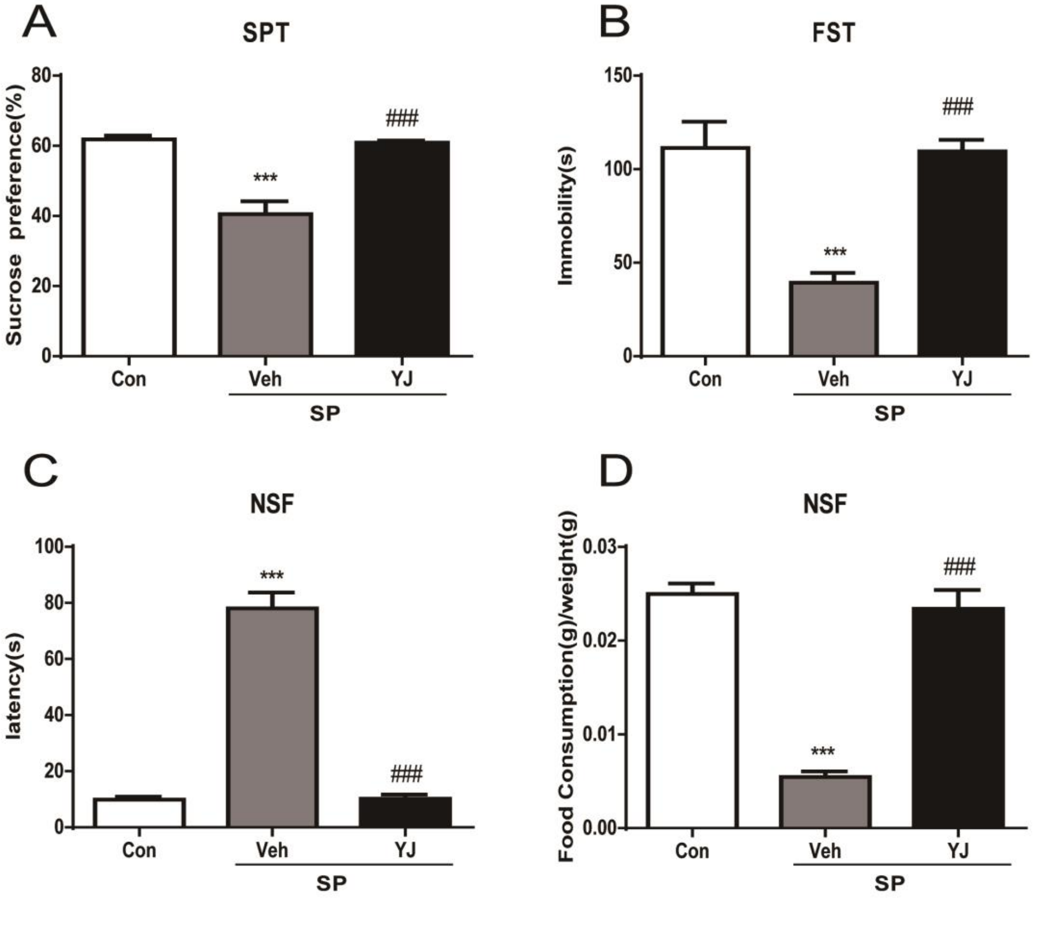

Supplement: Supplementary Information [file srep35042-s1.pdf]
